# Psychometric evaluation of an Adverse Childhood Experiences (ACEs) measurement tool: an equitable assessment or reinforcing biases?

**DOI:** 10.1186/s40352-022-00198-2

**Published:** 2022-11-29

**Authors:** Xiaohan Mei, Jiayu Li, Zhi-Shu Li, Shun Huang, Li-Li Li, Yang-Hong Huang, Jianhong Liu

**Affiliations:** 1 California State University, Los Angeles, 5151 State University Dr, Los Angeles, CA 90032 USA; 2 Washington State University, Pullman, WA 99164-2520 USA; 3 Shenyang Open University, Shisiwei Rd, Heping District, Shenyang, Liaoning 110003 China; 4 Beijing Hospital, 1 Dahua Rd, Dongcheng, Beijing, 100051 China; 5 Shenyang Maternity and Child Health Hospital, 41 Shenzhou Street, Shenhe District, Shenyang, 110000 China; 6 Shenyang Women and Children’s Hospital, No. 87 Danan street, Shenhe District, 110000 Shenyang, China; 7 University of Macau, Avenida da Universidade Taipa, Macau, 999078 China

**Keywords:** Adverse childhood experiences (ACEs), Structural validity, Measurement invariance, Group Comparison

## Abstract

**Background:**

Utilizing Adverse Childhood Experiences (ACEs) measurement scales to assess youths’ adversities has expanded exponentially in health and justice studies. However, most of the ACEs assessment scales have yet to meet critical psychometric standards, especially for key demographic and minority groups. It is critical that any assessment or screening tool is not reinforcing bias, warranting the need for validating ACEs tools that are equitable, reliable and accurate. The current study aimed to examine the structural validity of an ACEs scale. Using data from the 2019 Behavioral Risk Factor Surveillance System (BRFSS), which collected of 97,314 responses collected from adults across sixteen states. This study assessed the psychometric properties and measurement invariance of the ACEs tool under the structural equation modeling framework.

**Results:**

We found the 11-item ACEs screening tool as a second-order factor with three subscales, all of which passed the measurement invariance tests at metric and scalar levels across age, race, sex, socioeconomic status, gender identity, and sexual orientation. We also found that minority groups experienced more childhood adversity with small effect size, with the exception of the gender identity.

**Conclusion:**

The ACEs measurement scale from the BRFSS is equitable and free from measurement bias regardless of one’s age, race, sex, socioeconomic status, gender identity, and sexual orientation, and thus is valid to be used to compare group mean differences within these groups. The scale is a potentially valid, viable, and predictive risk assessment in health and justice and research settings to identify high-risk groups or individuals for treatments.

## Background/Rationale

A relatively recent public health and justice concept, the adverse childhood experiences (ACEs) scale, (Anda et al., 2010; Ford et al., 2020), is defined as “potentially traumatic events that occur in childhood (Centers for Disease Control and Prevention, 2022).” The American Academy of Pediatrics’ (AAP’s) policy statement encourages pediatricians to screen ACEs for the toxic stress of children and adolescents early (Committee on Psychosocial Aspects of Child and Family Health et al., 2012). ACEs have been found to be associated with increased physical and mental illness through the engagement of health-risk behaviors (Baldwin et al., 2021a; Centers for Disease Control and Prevention, 2022; Hughes et al., 2021), and has been linked to $748 billion in related health costs (Bellis et al., 2019). Recently, the COVID-19 pandemic worsened the youths’ ACEs and toxic stress (Ortiz et al., 2022), as counties in the world implemented lockdown policies, closed schools, and disrupted governmental and private services, which left many children unprotected. Several countries started implementing ACEs screening through either universal (e.g., well-childcare) or targeted platforms (e.g., pediatricians), but the ACE-induced health issues are unlikely to be neutralized without the appropriate treatments and interventions; however, the limited studies suggested that screening for ACEs improves adversity identification and receiving community-based services.

### ACE assessment, structural validity and measurement invariance/bias

The bridge between adversity identification, risk assessment and intervention/treatment referral or resource allocation is the ACEs screen and assessment tools (Gordon et al., 2020). Despite the utility of the ACE assessment or screening, no instrument has accumulated sufficient psychometric evidence to demonstrate its superiority in terms of its predictive accuracy and economic viability^1^ (Loveday et al., 2022). Despite its utility, there are many methodological concerns of the ACEs assessment remains to be resolved (Holden et al., 2020). One and the most fundamental methodological concern is the how well ACEs are assessed or the validity of the assessment itself (Holden et al., 2020). This concern is two-fold. First, “what is the underlying factor structure of childhood adversities?” and second, “does the instrument demonstrate measurement invariance,” or “is the instrument equally appropriate for assessing adversity from a variety of individuals? (Holden et al., 2020, p.169)”

While the first question pertains to the ACEs assessment of structural validity, the second question deals with measurement bias imbedded in the assessment instrument itself, which would produce biased estimation for key demographic groups. While there are twenty different versions of the ACEs assessment scales, ranging from 8 to 70 items per instrument, only four studies explored the structural validity of the ACEs instruments, among which only three studies investigated the measurement invariances/bias across certain demographic groups, such as age and sex (see Holden et al., 2020).

Briefly, when evaluating the measurement invariance, researchers must provide at least three levels or tiers (configural, metric and scalar) of evidence to claim the assessment instrument is not biased toward any of the subgroups (Ford et al., 2014). Nevertheless, one the of ACEs assessment scale validation study claimed that they achieved measurement invariance, but in fact the measurement invariance failed at the scalar level for youth gender groups (girls and boys) (Meinck et al., 2017). The other two studies tested and passed the measurement invariance of two different version of ACEs scales (from the Panel Study of Income Dynamics and the BRFSS project) across gender and age (Olofson, 2018; Ford et al., 2014). Unfortunately, the equality of the assessment based on ones’ group memberships, especially for social disadvantaged minority groups yet to be tested and validated (i.e., sexual and gender minorities). There is evidence that disadvantaged, or minority groups are more likely to suffer various types of early childhood adversities (Centers for Disease Control and Prevention, 2022). Although disadvantaged groups might score higher on the ACEs assessment scales, the differences found between disadvantaged and non-disadvantaged groups might be artificial and could only be the product of measurement biases imbedded in the assessment itself due to the lack of measurement invariance validations.

### Health equity tourism

Since the seminal research on ACEs (Felitti et al., 1998), the ACEs studies on justice and health outcomes have proliferated (Baglivio et al., 2014). Researchers recognized that the interrelationship between the ACEs and social inequalities (McEwen & Gregerson, 2019; Racine et al., 2022). However, in order to address the ACEs among the youth’s population through preventive measures, both the clinicians and researchers must have the assessment tool to accurate measure the underlying ACEs constructs across all key demographic groups.

Lett and colleges defined ‘health equity tourism’ as researchers jumped on the bandwagon of equity research for pursuing health and justice publications or fundings without investigating the sources of the resource of inequality (i.e., structural racism) (2022). The ACEs research to date have not resolve the measurement methodology issues (i.e., measurement invariance) for ACEs assessment, which questions the validity of some research findings of all ACEs studies, especially for minority groups (Holden et al., 2020). Without empirical evidence that an ACE assessment scale is unbiased across disadvantaged groups, the ACE assessment might, instead of addressing and improving, reinforce social inequality because the assessment contents and items might be inappropriate to assessing adversity for the minority groups.

### Current study

Therefore, to fill the gap in the literature regarding the lack of critical evaluation of the ACEs assessment scales used by researchers and clinical professionals, we attempt to investigate the structural validity of the ACEs scale from the BRFSS while evaluating the measurement bias for the vulnerable and marginalized populations, such as racial ethnic minorities, people with lower socioeconomic status, sexual and gender minorities. We select the ACEs scale from the BRFSS because it is one of the few promising instruments that has accumulated considerable psychometric evidence (Holden et al., 2020). This instrument can also be used in a self-report format, which has been used to generate a national representative sample to obtain external validity. This instrument economic with only has 11 items, which can be easily to be adopted and incorporated into many research projects without burden the participants. Therefore, in this study we attempt to validate the structural validity and the lack of measurement bias of this ACEs assessment scale from the BRFSS with a national representative sample. We hypothesized that this ACEs assessment scale are free from measurement biases across all major minority groups.

## Methods

### Study sample

In this study, we evaluated the internal latent structure (structural validity) and the measurement bias of the ACEs scale using data from the 2019 Behavioral Risk Factor Surveillance System (BRFSS; http://www.cdc.gov/brfss/). The BRFSS was initiated by the Centers for Disease Control and Prevention (CDC) in 1984. According to the BRFSS Data User Guide (2013), state health departments, assisted by the CDC, conducted yearly telephone surveys to collect data with standard protocols on adults’ risk behaviors, preventive health practices, and health status. For each year, the annual sample contains more than 4,000 telephone interviews that were conducted for each state. The BRFSS used a stratified random sampling approach with a weighting protocol, ensuring the generalizability and representativeness of many demographic characteristics, such as sex, age, race and education. We used the 2019 BRFSS sample (*N* = 97,314) from the BRFSS, which collected ACEs assessments from sixteen states, including Alabama, Delaware, Florida, Indiana, Iowa, Michigan, Mississippi, Missouri, New Mexico, North Dakota, Pennsylvania, Rhode Island, South Carolina, Tennessee, Virginia, West Virginia, and Wisconsin. The sample characteristics are reported in Table 1.

**Table 1:** Sample descriptive (*N* = 97,314)

	Mean (S.D.) /*Percentage*	Missing (*%*)
**Adverse Childhood Experiences (ACEs)**	2.35 (3.20)	.0
* Household Dysfunction*	.79 (1.13)	.0
* Emotional/Physical Abuse*	1.25 (1.82)	.0
* Sexual Abuse*	.38 (1.20)	.0
* ACEs Summary Score*	2.35 (3.20)	.0
**Sex**		.0
* Male*	*45.4*	
* Female*	*54.6*	
**Age**		2.1
* 18–24*	*6.0*	
* 25–34*	*10.5*	
* 35–44*	*11.8*	
* 45–54*	*14.6*	
* 55–64*	*20.1*	
* 65+*	*37.0*	
**Income**		*19.1*
* Less than 15,000*	*9.1*	
* 15,000 to less than 25,000*	*16.2*	
* 25,000 to less than 35,000*	*10.7*	
* 35,000 to less than 50,000*	*14.3*	
* 50,000+*	*49.7*	
***Race***		2.1
* White only, Non-Hispanic*	*75.9*	
* Black only, Non-Hispanic*	*7.7*	
* Other race only, Non-Hispanic*	*5.2*	
* Multiracial, Non-Hispanic*	*2.1*	
* Hispanic*	*9.1*	
**Sexual Orientation**		43.9
* Straight*	*95.4*	
* Others*	*4.6*	
**Gender Identify**		43.6
* Transgender*	*.4*	
* Not Transgender*	*99.6*	

## Measurements

The outcome measure is the ACEs, which contains eleven binary and ordinal items assessing whether an individual suffered various types of adverse childhood abuses, such as physical, verbal, and sexual abuse, as well as experienced any traumatic events, such as the parental incarceration and separation. The full item descriptive statistics were reported in Table 2.

**Table 2:** ACEs Item descriptive statistics (*N* = 97,314)

Item	Frequency	*%*	λ	Missing%
1. Household Dysfunction				
1. Live with anyone depressed, mentally ill, or suicidal	--	--	.731	.0
* No*	81,927	*84.2*	--	--
* Yes*	15,387	*15.8*	--	--
2. Live with a problem drinker/alcoholic?	--	*--*	.731	.0
* No*	75,539	*77.6*	--	--
* Yes*	21,775	*22.4*	--	--
3. Live with anyone who used illegal drugs or abused prescription?	--	*--*	.731	.0
* No*	91,194	*91.3*	--	--
* Yes*	6,120	*8.7*	--	--
4. Live with anyone who served time in prison or jail?	--	*--*	.731	.0
* No*	91,194	*93.7*	--	--
* Yes*	6,120	*6.3*	--	--
5. Were your parents divorced/separated?	--	*--*	.731	.0
* No*	73,975	*76.0*	--	--
* Yes*	23,339	*24.0*	--	--
2. Emotional/Physical Abuse				
6. How often did your parents beat each other up?	--	*--*	.827	.0
* Never*	82,565	*84.8*	--	--
* Once*	3,601	*3.7*	--	--
* More than once*	11,148	*11.5*	--	--
7. How often did a parent physically hurt you in any way?	--	*--*	.827	.0
* Never*	75,888	*78.0*	--	--
* Once*	5,666	*5.8*	--	--
* More than once*	15,760	*16.2*	--	--
8. How often did a parent swear at you?	--	*--*	.827	.0
* Never*	67,600	*69.5*	--	--
* Once*	4,978	*5.1*	--	--
* More than once*	24,736	*25.4*	--	--
3. Sexual Abuse				
9. How often did anyone ever touch you sexually?	--	*--*	.952	.0
* Never*	86,954	*89.4*	--	--
* Once*	3,821	*3.9*	--	--
* More than once*	6,539	*6.7*	--	--
10. How often did anyone make you touch them sexually?	--	*--*	.952	.0
* Never*	89,731	*92.2*	--	--
* Once*	2,880	*3.0*	--	--
* More than once*	4,703	*4.8*	--	--
11. How often did anyone ever force you to have sex?	--	*--*	.952	.0
* Never*	92,961	*95.5*	--	--
* Once*	1,538	*1.6*	--	--
* More than once*	2,815	*2.9*	--	--

When testing ACEs’ measurement bias, we used six nominal grouping variables, including age, race, sex, socioeconomic status, sexual identity, and sexual orientation. Age was operationalized into six categories, including “18–24,” “25–34,” “35–44,” “45–54,” “55–64” and “65+.” The biological sex was operationalized as either “male” or “female.” Income was operationalized six categories, including “less than 15,000,” “15,000 to less than 25,000,” “25,000 to less than 35,000,” “35,000 to less than 50,000,” “50,000+,”^2^ and “Don’t know/Not sure/Missing.” Race was operationalized into five categories, including “white only”, “non-Hispanic,” “black only, non-Hispanic,” “other race only, non-Hispanic,” “multiracial, non-Hispanic,” “Hispanic.” Sexual orientation is measured as “straight” and “others”, which include gay, bisexual, something else, and I don’t know the answer. Sexual identity was measured as “not transgender” and “transgender.”

## Analytical Strategy

We first conducted an Exploratory Factor Analysis (EFA) to discover the underlying factorial pattern. Second, we conducted a sequential Multi-group Confirmatory Factor Analysis (MGCFA) to confirm the suggested factorial pattern. We extracted a second-order factor through higher-order modeling when we identified that the factors shared a substantial amount of common variance (Chen et al., 2005; Putnick & Bornstein, 2016). Once the internal latent structure of the ACEs was identified, we tested three essential forms of measurement bias or invariances, including configural, metric, and scalar, across all the group memberships (Schmitt & Kuljanin, 2008). Moreover, we reported the latent mean difference (i.e., true mean difference) represented by the Cohen’s *d* (Fritz et al., 2012) across all six group memberships. We followed the interpretations provided by Cohen (Cohen, 1988) when evaluating the effect size of the mean difference, ranging from small (0.20), medium (0.50), and large (0.80) effect size. In addition, we performed a common factor model when measurement invariance was achieved at all three invariance levels.

We followed guidelines for testing sequences of measurement invariance and higher-order factors (Chen et al., 2005; Rudnev et al., 2018). The fixed factor approach was used and we followed the model specification and identification suggestions by previous studies (Byrne & Stewart, 2006; Millsap & Yun-Tein, 2004). We performed omnibus tests for higher-order modeling and measurement invariance tests and conducted further testing when the omnibus tests failed (Little, 2013). The Weighted Least Square Mean and Variance Adjusted (MLSMV) is the preferred estimator because the items are categorical/ordinal and polytomous. The ‘Theta’ parameterizations is selected because it allowed us to test all forms of measurement invariances (Muthén & Asparouhov, 2002). Because the items are categorical, we conducted all tests within the Item Factor Analysis (IFA)/Item Response Theory (IRT) framework (Thomas, 2011). The missing data are handled with the full information maximum-likelihood (FIML) approach with MLSMV estimator when there is non-substantial missing at random data (Asparouhov & Muthen, 2010). The FIML is a superior method than the listwise deletion, pairwise deletion and imputation approaches (Enders & Bandalos, 2001).

Next, we computed the coefficient omega (ω) to evaluate the construct reliability of the G-factor and subscales. Using the Omega coefficient is advantageous over Cronbach’s Alpha because it assumes a parallel construct measurement structure (Deng & Chan, 2017; Geldhof et al., 2014; Nájera Catalán, 2019) and it enables researchers to accurately evaluate the construct reliability for higher-order factors (Nájera Catalán, 2019). A threshold of 0.65 for multidimensional (higher-order) and 0.80 for unidimensional (first-order) measures were used as thresholds to determine the ‘acceptable’ level of construct reliability (Nájera Catalán, 2019).

When evaluating the goodness of the EFA model, we followed the industry standard which considers both theory and the empirical evidence, such as the Kaiser-Guttman rule and goodness of fit, to determine the number of factors (Brown, 2015). For item loadings and cross-loadings, we also followed Comrey and Lee’s (Comrey & Lee, 1992) guidelines that the strength of the loadings and cross-loadings range from poor (.32), fair (.45), good (.55), very good (.63) or excellent (.71) fit. When evaluating the goodness of the CFA models, we compared item and factor loadings/cross-loadings with industry-standard loading thresholds of poor (.32), fair (.45), good (.55), very good (.63), and excellent (.71) (Tabachnick et al., 2007). Model fit is ‘acceptable’ if the Comparative Fit Index (CFI)/Tucker Lewis Index (TLI) are equal or greater than .90 and the Root Mean Square Error of Approximation (RMSEA) is equal/less than .08. The model fit is ‘good’ when CFI/TLI are equal or exceed .95 and the RMSEA is equal/less than .05 (Brown, 2015; Little, 2013). Models were evaluated with constraints added to each additional and progressive model for higher-order and group invariance tests. Higher-order models and those with additional measurement invariance constraints were retained if the ∆CFI and ∆TLI values were equal/less than .01, indicating that the nested higher-order modeling or additional measurement invariance constraints did not produce any detrimental effect on the models (Little, 2013).

## Results

We identified that the ACEs scale was a second-order model with three subscales. The EFA suggested a two-factor model because there were two Eigenvalues above 1, yet the SRMR model fit was not ideal (CFI = .985, TLI = .976, RMSEA = .027, SRMR = .055). Also, compared with a 2-factor model, the 3-factor model made significant improvement in all models’ fit indices with ∆CFI and ∆TLI above .10 (CFI = .997, TLI = .993, RMSEA = .015, SRMR = .023). The assessment content is aligned with the suggested factorial pattern in the 3-factor model, assessing three sub-types of ACEs: *household dysfunction*, *emotional/physical abuse*, and *sexual abuse*.

**Fig. 1:**
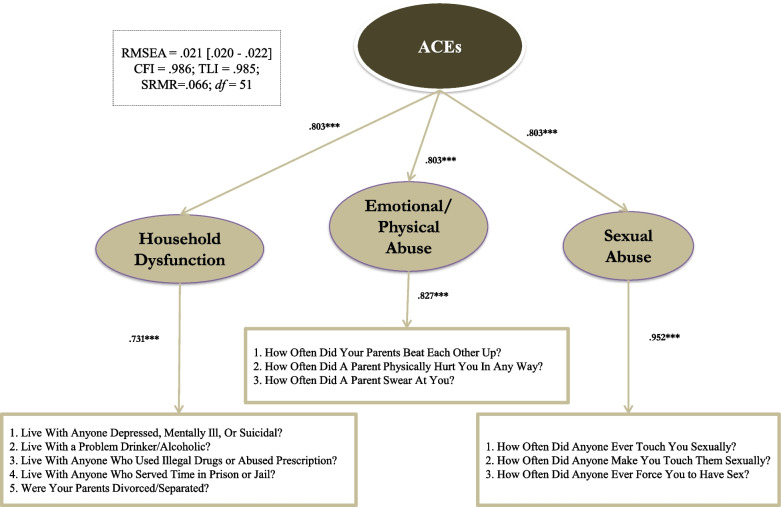
Final model of Adverse Childhood Experiences (ACEs)

Next, we retained the three-factor model and subjected the measurement model to measurement invariant tests and higher-order model tests. As a result, we successfully exacted a second-order model, as the second-order model did not produce detrimental model fits (with ∆CFI and ∆TLI below .010) compared to the measurement model across all six grouping models. Also, as shown in Table 3, the second-order model passed all three levels of invariances (i.e., configural, metric, and scalar) for all six groups as the ∆CFI and ∆TLI did not exceed .10 for all models (Table 4). Finally, we conducted a common factor model, combing all the groups, and the final model fits exceeded all thresholds to be at least considered “acceptable” (CFI = .986, TLI = .985, RMSEA = .021, SRMR = .066). The reliability of the ACE scale reached an acceptable level of reliability, which passed the threshold of .65 for multidimensional measures (ω = .906). We provided a visual illustration of the ACE final model in Fig. 1.

**Table 3:** Measurement invariance tests across age, race, sex, income, sexual identity, and sexual orientation groups

Model^a^	Tests of Invariance & Structure	*df*	CFI	TLI	RMSEA [90% C.I.]	SRMR	Δ *df*	Δ CFI	Δ TLI
**A. Across Age Groups**									
** A1**	Measurement model	246	0.994	0.992	.017 [.016 − .018]	.044	--	--	--
** A2**^**a**^	Second-order model/configural invariance	258	0.988	0.985	.024 [.023 − .025]	.062	12	.006	.007
** A3**^**b**^	First-order within-scale parallel model	306	0.984	0.983	.026 [.025 − .026]	.073	48	.004	.002
** A4**	First- and second-order metric invariance	321	0.984	0.984	.025 [.024 − .026]	.075	15	.000	+ .001
** A5**	First- and second-order scalar invariance	401	0.981	0.984	.024 [.024 − .025]	.078	80	.003	.000
**B. Across Race Groups**									
** B1**	Measurement model	205	0.994	0.992	.015 [.014 − .016]	.037	--	--	--
** B2**^**a**^	Second-order model/configural invariance	215	0.986	0.982	.023 [.022 − .023]	.057	10	.008	.010
** B3**^**b**^	First-order within-scale parallel model	255	0.983	0.982	.022 [.022 − .023]	.069	40	.003	+ .000
** B4**	First- and second-order metric invariance	267	0.984	0.984	.022 [.021 − .022]	.070	12	+ .001	+ .002
** B5**	First- and second-order scalar invariance	331	0.983	0.986	.020 [.019 − .021]	.071	64	.001	+ .002
** C. Across Sex Groups**									
** C1**	Measurement model	82	0.994	0.992	.016 [.015 − .017]	.038	--	--	--
** C2**^**a**^	Second-order model/configural invariance	86	0.987	0.984	.023 [.022 − .023]	.059	4	.007	.008
** C3**^**b**^	First-order within-scale parallel model	102	0.985	0.984	.022 [.021 − .023]	.069	16	.002	+ .000
** C4**	First- and second-order metric invariance	105	0.985	0.985	.022 [.021 − .022]	.070	3	+ .000	+ .001
** C5**	First- and second-order scalar invariance	121	0.984	0.986	.021 [.020 − .022]	.071	16	.001	+ .001
**D. Across Income (SES) Groups**									
** D1**	Measurement model	205	0.993	0.991	.018 [.017 − .019]	.040	--	--	--
** D2**^**a**^	Second-order model/configural invariance	215	0.984	0.980	.026 [.025 − .027]	.062	10	.009	.011
** D3**^**b**^	First-order within-scale parallel model	255	0.980	0.979	.027 [.026 − .028]	.074	40	.004	.001
** D4**	First- and second-order metric invariance	267	0.981	0.981	.026 [.025 − .026]	.074	12	+ .001	+ .002
** D5**	First- and second-order scalar invariance	331	0.981	0.984	.023 [.022 − .024]	.075	64	.000	+ .003
**E. Sexual Identity Groups**									
** E1**	Measurement model	82	0.995	0.993	.013 [.012 − .014]	.037	--	--	--
** E2**^**a**^	Second-order model/configural invariance	86	0.988	0.984	.019 [.018 − .020]	.057	4	.007	.009
** E3**^**b**^	First-order within-scale parallel model	102	0.987	0.986	.018 [.017 − .019]	.068	16	.001	+ .002
** E4**	First- and second-order metric invariance	105	0.988	0.987	.018 [.017 − .019]	.068	3	+ .001	+ .001
** E5**	First- and second-order scalar invariance	121	0.988	0.989	.016 [.015 − .017]	.068	16	.000	+ .002
** F. Sexual Orientation Groups**									
** F1**	Measurement model	82	0.996	0.994	.013 [.012 − .015]	.039	--	--	--
** F2**^**a**^	Second-order model/configural invariance	90	0.990	0.987	.020 [.019 − .021]	.061	8	.006	.007
** F3**^**b**^	First-order within-scale parallel model	102	0.988	0.987	.021 [.020 − .022]	.069	12	.002	.000
** F4**	First- and second-order metric invariance	105	0.987	0.987	.021 [.020 − .022]	.070	3	.001	.000
** F5**	First- and second-order scalar invariance	121	0.988	0.989	.019 [.018 − .020]	.070	16	+ .001	+ .002
**G. Common Factor Model**									
** G1**	Measurement model	41	0.993	0.991	.017 [.016 − .017]	.036	--	--	--
** G2**^**a**^	Second-order model	43	0.987	0.983	.023 [.022 − .023]	.056	2	.006	.008
** G3**^**b**^	First-order within-scale parallel model	51	0.986	0.985	.021 [.020 − .022]	.066	8	.001	+ .002

^a^First-order factor loadings were set to be equal within groups to obtain an over-identified model

^b^First-order within-factor items are constrained to be equal but allowed to vary across groups

**Table 4:** Latent mean difference for ACEs across age, race, sex, income, sexual identity, and sexual orientation

Group Memberships	Comparison Groups	Latent Mean Difference	*p-value*	SD_pooled_	Effect Size (*d*)
**Age**	18–24^a^	--	--	--	--
	25–34	.04	.29	3.64	.01
	35–44	− .05	.17	3.64	.01
	45–54	− .07	.**03**	3.55	.02
	55–64	− .15	**< .001**	3.35	.04
	65+	− .43	**< .001**	3.00	0.14
**Race**	White only, Non-Hispanic^a^	--	--	--	--
	Black only, Non-Hispanic	.26	**< .001**	3.11	.08
	Other race only, Non-Hispanic	.06	0.41	3.38	.02
	Multiracial, Non-Hispanic	.55	**< .001**	3.74	.15
	Hispanic	.20	**.001**	3.34	.06
**Sex**	Male^a^	--	--	--	--
	Female	.24	**< .001**	3.16	.08
**Income**	Less than 15,000^a^	--	--	--	--
	15,000 to 25,000	− .14	**.005**	3.77	.04
	25,000 to 35,000	− .26	**< .001**	3.63	.07
	35,000 to 50,000	− .29	**< .001**	3.58	.08
	50,000+	− .41	**< .001**	3.47	.12
**Sexual Identity**	Not Transgender^a^	--	--	--	--
	Transgender	.21	0.19	3.34	.06
**Sexual Orientation**	Straight^a^	--	--	--	--
	Others	.69	**< .001**	3.75	.18

^a^The Reference Group

Now, we present the true score differences across six group memberships in Table 4. We found that compared people aged between 18 and 24, people aged between 45 and 54 (*d* = .02, *p* < .05), 55 and 64 (*d* = .04, *p* < .001), and people who are above the age of 65 (*d* = 0.14, *p* < .001) reported statistically lower ACEs scores. Compared to non-Hispanic whites, Black only (*d* = .08, *p* < .001), non-Hispanic multiracial (*d* = .15, *p* < .001), and Hispanic (*d* = .06, *p* < .001) scored significantly higher. Females scored higher on ACEs than male participants (*d* = .08, *p* < .001). Compared to people whose income was less than $15,000, people in higher-income groups scored significantly lower ACEs (*d* = .04 − .16, *p* < .001). Compared to heterosexual people, sexual minorities scored significantly higher (*d* = 0.18, *p* < .001). Gender minorities (i.e., people who identified as transgender) scored higher than people who are cisgender (*d* = .18, *p* > .05), yet the mean difference is not statistically significant. With Cohen’*d* less than 0.20, all the statistical differences we found were small.

## Discussion

The current study made several contributions. First, consistent with a previous study that used an early version of the BRFSS data (D. C. Ford et al., 2014), we found the CDC’s ACEs Scale contains three subscales, including *household dysfunction*, *emotional/physical abuse*, and *sexual abuse*. Compared to the ACEs total score, each of its subscales has fewer items and, therefore, less variation and range. We advocate for using the composite scores of the ACEs scale with all items for screening instead of using three subscales separately because the common variance of the three subscales can be explained by one underlying factor, namely the ACEs, through second-order modeling. Given each of the three subscales has a limited number of items and range, and the utility of the subscales is yet to be fully explored, greater weight should be given to the entire ACE assessment in clinical practice for screening and public health research. Once the screening is completed, clinical practitioner could use more extensive and comprehensive tools to fully assess youths’ ACEs, and which type or subtype of the ACEs is the most stressful and traumatic for the youths. The finding of the current study demonstrated the ACEs assessment instrument can provides the clinicians a potentially promising, viable and economic screening tool to assess ACEs.

Second, the ACE scale passed the three levels of invariance tests (i.e., configural, metric, and scalar) across six group memberships, indicating that the ACEs assessment is equitable and free from measurement bias regardless of one’s age, race, sex, socioeconomic status, sexual identity, and sexual orientation. In other words, the ACEs scale is a valid screening tool to assess the group mean differences within these groups.

Third, since the ACEs scale is invariant, we used it to examine the group differences in age, race, gender, income, sexual identity, and sexual orientation. We found evidence suggesting that as one’s age increases, their ACEs scores decrease, such as significant relationship no longer holds for people were 45 and older. Given that the data were collected through the participants’ memory, there was an increased risk of recall bias for people aged 45 and older, suggesting that using the ACEs might not be suitable for clinical and research use if the individuals are older than 45-years-old because of the recall bias.

Furthermore, previous studies on group differences, such as gender, racial, and sexual minorities group differences, in ACEs often examine different types of ACEs separately (Andersen & Blosnich, 2013; Fang et al., 2016; Lee & Chen, 2017), and this study filled this gap by examining group differences in the ACEs as a single construct. Consistent with the previous findings, we found that non-Hispanic black, Hispanic, and non-Hispanic multiracial people reported higher ACEs, which indicated that people of racial minority experienced more adverse childhood experiences than white people. Similar to a previous study that females were at more risk of multiple types of ACES (Fang et al., 2016), females in this study reported higher ACES than males. In addition, we found that people’s socioeconomic status is significantly and negatively associated with ACEs.

Moreover, gender minorities reported higher ACEs than people who are cisgender. However, such a relationship is not statistically significant. Also, sexual minorities scored higher than heterosexual people. A possible explanation is that the difference and disparity can be attributed to structural racism (Dougherty et al., 2020). Alternatively, multi-level (micro and macro) and multisystem (family and neighborhood) characteristics could also explain said disparities. Unfortunately, without adequately designed research, the challenges of explaining the health disparity cannot be properly investigated in the current study (Jeffries et al., 2019).

We found that the effect sizes of the reported group differences are small. Overall, the findings support the theory that the vulnerable population, including women, young adults, racial ethnic minorities, people lower on the socioeconomic ladder, and LGBT groups, suffered more adverse, traumatic, physical, psychological, and sexual abuses in their early lives. Due to the limited scope of this research, we did not examine the intersectionality of the disadvantaged groups could experience more ACEs. Given the current finding, it is reasonable to speculate that the youths belong to multiple disadvantaged groups could have experiences more ACEs than non-disadvantaged population.

The current public, justice and health system might not have the capacity to address the needs for all individuals (McLennan et al., 2020). Fortunately, with this validated ACEs, it is possible to accurately identify these high-risk vulnerable individuals. Also, the traditional prevention strategy framework recognizes that children with higher risk should be prioritized to receive prevention treatments (Brennan et al., 2020). The traditional prevention strategy often consider race, sex, sexual orientation, sexual identity, socioeconomic status and age independently and therefore fails to address the multiple intersecting needs of the individuals (Qureshi et al., 2022). While the finding of this research calls for critical examination of the underlying structure and factors that contributed to the disparities and how the prevention programs could be tailored to multiple intersecting higher-than-average needs of the minority populations who are likely belong multiple disadvantaged groups.

## Limitations

The current study has several limitations. First, not all states collected data on ACEs in the 2019 study. Although the sample is large, the generalizability to the entire U.S. population of the findings remains to be further validated. Even with more states’ participation, the generalizability of the result is still confined to the U.S. and North America. Second, due to the limited scope, the predictive accuracy of the CDC’s (11-item) version of the ACEs (both the composite total and subscales’ score) remains to be further tested in future research across various justice and health outcomes as well as across various groups of children as the previous research demonstrated the lack of prediction precision for health issues (Baldwin et al., 2021b). Future research could maximize the ACEs predictive accuracy by using more sophisticated weighting schemes based on its empirical relationship with various health outcome interests to further support the screening practices (Holden et al., 2020). Future research could use longitudinal instead of cross-sectional data to validate the precision of the ACE assessment when used to predict or explain justice or health outcomes, such as illegal substance abuse, chorionic disease, and mental health. Researchers may even consider a more complicated model to account for the mediation or neutralizing effect of the positive childhood experience on ACEs (Ortiz et al., 2022).

Third, the data probably underestimated the prevalence of ACEs especially for sexual minorities because of the housing insecurity or instability (Tran et al., 2022). It is difficult to estimate the prevalence of homeless because of the heterogeneity of the samples in previous studies. Yet, it is evidentiary that sexual minority in general at disproportionately higher risk of homeless (Corliss et al., 2011). Therefore, sexual minorities are likely underrepresented in the 2019 BRFSS which is a household sample. The low frequency of gender minority youths in our sample might produce the non-significant group mean difference between gender identity groups. Future research should reinvestigate the ACEs difference between such groups with larger samples.

Also, the data were collected from adults’ recollection of the memory and therefore further underestimated the prevalence, especially for the older population (Tran et al., 2022). Next, the utility of the CDC’s ACEs screen instrument’s forecasting utility remains to be validated among the youth and children’s populations. Hence, longitudinal research tracking youths’ health development over time might offer more definitive evidence (Lacey et al., 2022). In addition, this study used EFA to identify subscale of the ACE and used (Multigroup) CFA to confirm the ACEs constructs with the same sample. Although the results are unlikely to differ from the current finding, future research could further revalidate the ACEs scale from the BRFSS with new data.

Last, researchers have identified that most of the current ACEs (including CDC’s version) did not follow the scale creation processes and standards and therefore lacked construct validity. Although the current research offered convincing evidence to support the potential utility of using it as a screening instrument, it still lacks content validity because of the limited items (*n* = 11) and measured types of adversity (Holden et al., 2020). While using existing tools with more screening content (Brennan et al., 2020), or expanding the content validity of the ACE might be beneficial, the CDC’s version of ACEs might be offered to health platforms as an economically viable screen tool, upon demonstrating its preferred level of prediction precision for various health outcomes. The limitation of the range and assessment content of the tool might be further mediated with more sophisticated psychometric methods, which allow clinicians and researchers to produce weighted latent factor scores (Grice, 2001). Applying more sophisticated methods from machine learning techniques might offer a sizeable boost to the predictive accuracy in clinical practices, which has been used in justice settings to predict health outcomes, such as substance abuse or drug crimes (Hamilton et al., 2021). Once the screen is completed and high-risk individuals are identified, a complete or a more comprehensive ACE clinical assessment might be employed to toxic stress risk (Harris, 2020).

## Data Availability

The datasets analyzed during the current study are available in the 2019 Behavioral Risk Factor Surveillance System (BRFSS; http://www.cdc.gov/brfss/).

## Footnotes

1 Economic viability is referred to as whether the tool is short but reliable and valid that can be used to assess ACEs in relatively cost-effective fashion.

2 We recognize this categorization which does not further distinguished people who are in higher socio-economic status (i.e., maybe 80,000 + or 150,000+). Unfortunately, this is the one of the limitations that this study which used a secondary data (i.e., the BRFSS) for analyses.
